# Confronting the connectivity crisis in human M/EEG research

**DOI:** 10.1016/j.tics.2025.09.001

**Published:** 2025-09-18

**Authors:** Ole Jensen, Hyojin Park, Oscar Ferrante

**Affiliations:** 1 Department of Experimental Psychology, University of Oxford, Oxford OX2 6GG, UK; 2 Oxford Centre for Integrative Neuroimaging, Oxford Centre for Human Brain Activity, Department of Psychiatry, University of Oxford, Oxford OX3 7JX, UK; 3 School of Psychology, Centre for Human Brain Health, University of Birmingham, Birmingham B15 2TT, UK; 4 School of Psychology, University of Surrey, Guildford GU2 7XH, UK

## Abstract

The cognitive neuroscience community using M/EEG has not converged on measures of task-related inter-regional brain connectivity that generalize across tasks and laboratories. We call for community-driven efforts to systematically test and validate connectivity metrics using shared datasets and protocols, aiming to establish robust, replicable frameworks for cognitive and clinical applications.

## The many measures of functional connectivity

**Figure 1:**
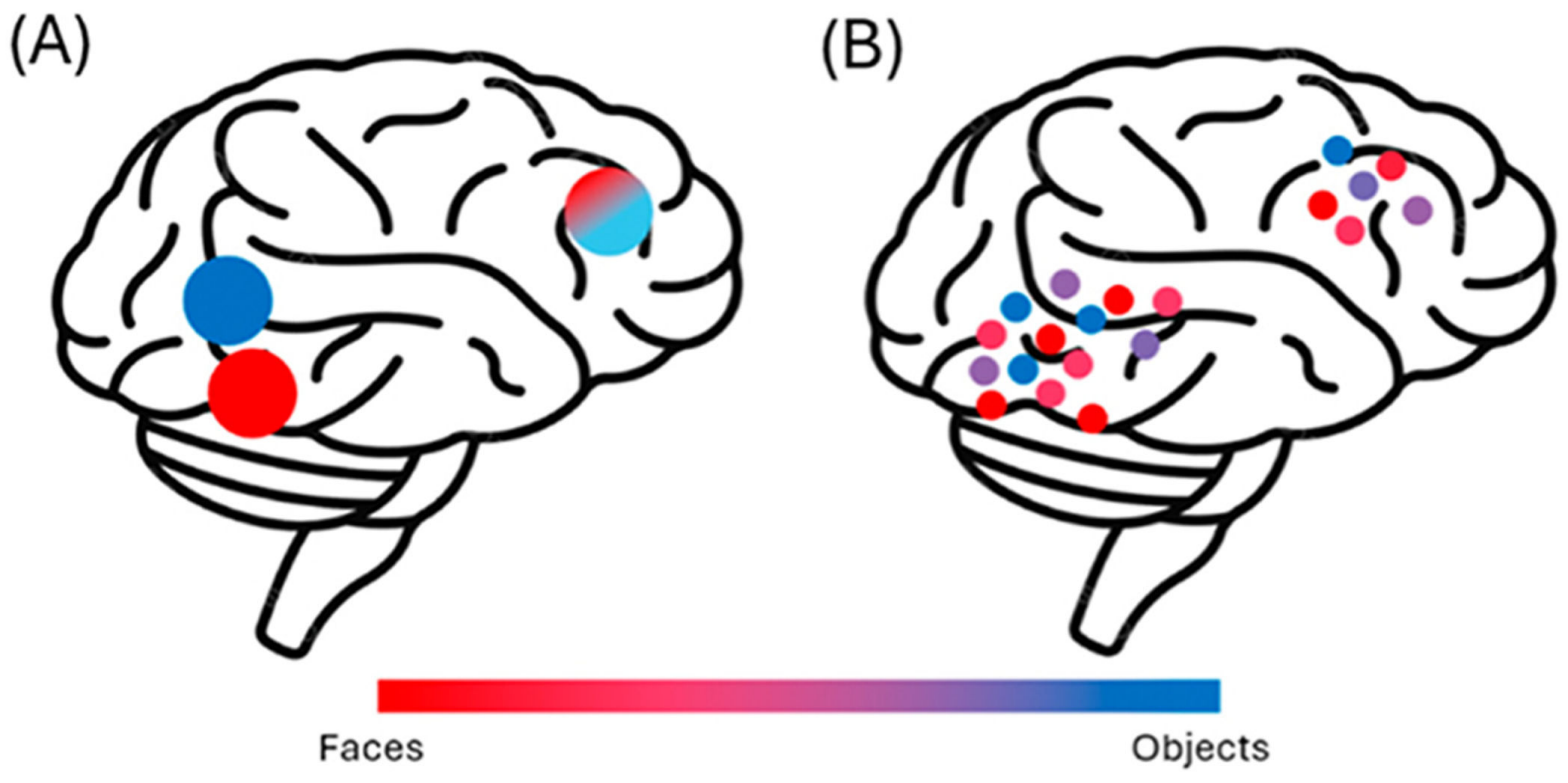
Investigating task-based functional connectivity in the human brain. The schematic examples depict putative networks supporting the perception of faces versus objects, which rely on communication between the ventral stream and the prefrontal cortex. (A) According to a modular view, faces and objects are represented by distinct regions in the temporal lobe. In this example, the communication between these regions and the prefrontal cortex facilitates the conscious perception of visually presented stimuli. While oscillatory interactions, for example, in the gamma band, have often been proposed to reflect task-modulated interareal functional connectivity [1,3,4], there are few findings based on data from electroencephalography (EEG) or magnetoencephalography (MEG) that replicate across tasks and laboratories. (B) A multivariate framework embraces distributed and overlapping representations in, for instance, the temporal and frontal lobes. Here, the time course in each region can be envisioned as a trajectory in the corresponding multidimensional space. The statistical dependence of these trajectories is then characterized [12]. While these multivariate measures are promising, replicated findings are scarce, and they must be benchmarked against other measures of connectivity.

The field of cognitive and clinical neuroscience has experienced significant advances over the past decades, particularly with the introduction of human brain imaging techniques. While considerable efforts have focused on identifying specific brain regions associated with particular tasks, it is widely accepted that brain regions do not operate in isolation, and it is imperative to study the brain as a network [1]. Consequently, the field has produced many promising tools for estimating task-dependent functional connectivity between brain regions of interest in electroencephalography (EEG) or magnetoencephalography (MEG) data (as exemplified in Figure 1A) [2]. This modular approach to inter-regional connectivity includes temporal correlations, **coherence** (see Glossary), **cross-frequency couplings**, and measures of **mutual information**.

However, because different research groups apply different measures for establishing long-range functional connectivity, cognitive neuroscientists have not converged on a commonly accepted approach. As a result, few human studies on taskbased functional connectivity have been replicated across laboratories, and these various methods are rarely benchmarked against each other. We therefore call for a systematic community-driven comparison of metrics of functional connectivity verified using benchmarked datasets with the aim of shifting the field from exploration towards consolidation.

## Functional connectivity measures relying on oscillatory phasic interactions

The measure of **phase synchronization** as an indicator of inter-regional functional connectivity was pioneered in the mid-90s and has since been formulated in several theoretical frameworks [1,3]. The **Communication Through Coherence (CTC)** theory outlines an explicit mechanism for communication between brain regions, suggesting that information exchange is supported when spikes from a sending oscillating population of neurons arrive at the excitatory oscillatory phase of neurons in a receiving population [3]. While the CTC framework has garnered support from various studies utilizing intracranial recordings in nonhuman primate visual regions, it has not yet generalized into a robust connectivity measure applicable to human EEG and MEG data beyond the early visual system.

Despite these limitations, many cognitive neuroscience theories still embrace the idea that inter-regional communication is supported by gamma phase synchrony. For instance, in an adversarial collaboration comparing the Global Neuronal Workspace Theory and Integrated Information Theory of consciousness, both frameworks predicted that inter-regional phase synchronization in the gamma band (30–90 Hz) would support conscious visual perception [4]. However, results showed that inter-regional phase synchronization was difficult to substantiate in a large MEG data set.

Oscillations in the theta (3–8 Hz), alpha (8–13 Hz) and beta (13–30 Hz) bands have also been suggested to reflect inter-regional communication [5–7]. While interactions in each of these frequency bands could support communication, the many approaches also highlight that the field has not yet reached a consensus on which frequency bands support connectivity and in which task contexts

## Functional connectivity measures not relying on oscillatory phasic interactions

Researchers have also explored the correlations in band-limited power fluctuations across different brain regions [8]. The approach has proven valuable for identifying resting-state networks in MEG data. Multiple groups have successfully identified and replicated the classical resting-state networks observed in studies using functional magnetic resonance imaging (fMRI) by analysing **power–power correlations** in the alpha and beta bands [8]; however, the approach has been rarely used to estimate task-based connectivity.

There is also a rich set of connectivity approaches based on **information-theoretical measures** [9]. The major advantage of information-theoretical measures is that they can capture both linear and nonlinear dependencies. Despite the promise and intuitive appeal of these approaches, they must be tested and applied more widely to assess how well the findings generalize.

### Dynamical causal modelling (DCM)

was initially developed to account for effective connectivity in fMRI data and was later adapted to quantify connectivity in EEG and MEG data [10]. While the DCM approach has provided interesting insights, it has also faced significant criticism. One major concern is the combinatorial challenge of selecting the correct model from numerous possibilities. Critics argue that if the number of models is not constrained, the model selection procedure may fail to recognize plausible models [11]. Although the DCM approach has produced interpretable results in EEG and MEG studies using, for example, the mismatch negativity (MMN) paradigm [10], the approach has not been widely adopted as a definitive method for functional connectivity, given the aforementioned concerns.

## Multivariate measures of connectivity

The absence of replicated ‘textbook examples’ in EEG and MEG studies on task-related functional connectivity between brain regions may point to a conceptual problem of the **modular view of brain functioning** in which a smaller number of circumscribed regions interact to support a given task. Rather, methods that embrace a distributed view of neuronal processing, whether applied to specific regions (Figure 1B) or fully distributed across the brain, may be more meaningful. Various **multivariate measures of connectivity** have indeed been proposed (e.g., [12]); however, the community has so far not converged on commonly agreed approaches that replicate across studies.

## Towards validated approaches for identifying robust measures of functional connectivity

The proliferation of metrics developed to quantify task-related functional connectivity in humans has introduced significant challenges, particularly due to the limited replication of findings across tasks and research groups. This methodological diversity has contributed to fragmentation within the field, with different laboratories employing different measures and focussing on different cognitive tasks. Consequently, cross-study comparisons are difficult, impeding the establishment of a unified framework for investigating functional connectivity. This lack of coherence not only restricts our capacity to elucidate the underlying neuronal network mechanisms but also hinders progress in understanding clinical disorders at the network level.

Here, we advocate for systematic, community-driven efforts to test methodologies on functional connectivity applied to human MEG and EEG data. This could involve adversarial collaborations in which proponents of specific connectivity measures agree upon a suitable dataset and criteria for evaluation, ideally involving blinded data for testing (Box 1). These efforts should be conducted using preregistered reports, making the success criteria explicit. To assess the generalizability of a given measure, it would be essential to evaluate the methods across datasets derived from different tasks. The success criteria for a given functional connectivity framework should include independent replication across laboratories as well as the explanatory power in the context of relevant cognitive neuroscience theories.

## Concluding remarks

Over the past three decades, the MEG and EEG research communities have developed a broad array of methods for quantifying task-based functional connectivity. Despite this methodological richness, there remains a lack of consensus on standardized approaches, and findings on task-based connectivity in humans are rarely replicated across different tasks and laboratories. To advance toward a robust and generalizable framework for functional brain connectivity, we advocate for a community-driven initiative in which connectivity measures are systematically evaluated through adversarial collaborations using standardized datasets and benchmarking criteria. Such an effort is essential for enabling replicable discoveries and fostering progress in both cognitive and clinical neuroscience by promoting a network-based understanding of brain function.

## Box 1 Towards benchmarking measures of functional connectivity.

We propose an open-science, community-wide initiative to systematically evaluate measures of functional connectivity using neuroimaging tools and datasets made publicly available. This effort would take the form of a collaborative, adversarial benchmarking framework involving several groups defining shared and pre-registered evaluation criteria. Key elements of the proposed framework include:

- Identification of participating groups, each advocating a specific measure of functional connectivity (e.g., coherence, information-theoretic, DCM, or multivariate approaches).
- Selection of task-based datasets with well-specified hypotheses (e.g., connectivity between region A and B is expected to differ between task conditions). Multiple paradigms involving tasks associated with different regions should be included.
- Partitioning of each dataset into a training set and a held-out, blinded test set (with task labels concealed).
- Preregistered methods for data preprocessing and analysis, along with agreement on pre-defined criteria for robustness checks (e.g., replicability, sensitivity to task contrasts, generalisability).
- Comparative evaluation and ranking of methods based on their performance on the blinded test sets (this could be done as an open competition hosted on, e.g., Kaggle).

Such an endeavour would require considerable coordination and likely the acquisition of additional data with adequate statistical power. We envision this effort being supported by joint funding mechanisms and executed in a spirit of open collaboration.

The outcome – whether it identifies a robust connectivity measure or reveals fundamental limitations in current methods – would have significant implications for the field. A positive result would establish a methodological foundation for future basic and translational research. A negative result would highlight the need for alternative – perhaps more distributed and high-dimensional – frameworks for capturing brain connectivity dynamics.

## Glossary

Coherence: a measure of phase consistency between signals from different brain regions over time, used as a measure of CTC of functional connectivity.
Communication Through Coherence (CTC): a theory suggesting that inter-regional brain communication is facilitated when oscillatory neural populations are synchronized, typically in the gamma band.
Cross-frequency coupling: the interplay between different frequency bands (e.g., alpha phase to gamma power coupling), which may support complex neural communication.
Dynamical causal modelling (DCM): a Bayesian framework for modelling and inferring the directed (causal) interactions among neuronal populations, where hidden neuronal dynamics are linked to observed brain signals through a generative model.
Information-theoretical measures: analytical tools (like mutual information) used to quantify how much information is shared between different brain signals, capturing both linear and non-linear dependencies.
Modular view of brain function: the idea that cognitive tasks are supported by interactions among a limited number of specialized brain regions.
Multivariate measures of connectivity: statistical approaches that assess interactions between multiple brain regions simultaneously, capturing distributed patterns of functional connectivity that cannot be explained by pairwise relationships alone.
Mutual information: a statistical measure from information theory. It is used to assess functional connectivity by measuring how much knowing the activity in one brain region reduces uncertainty about the activity in another.
Phase synchronization: the alignment of oscillatory phases between brain regions, thought to enable coordinated neural activity.
Power–power correlation: a connectivity measure based on correlations in signal power across brain regions, rather than phase relationships.
